# Epilepsy Care and Mental Health Care for People with Epilepsy: California Health Interview Survey, 2005

**DOI:** 10.5888/pcd9.110140

**Published:** 2012-02-23

**Authors:** Alexander W. Thompson, Rosemarie Kobau, Royce Park, David Grant

**Affiliations:** Behavioral Health Services, Group Health Cooperative. Through the course of this work, Dr Thompson has been affiliated with the University of Washington, Department of Psychiatry and Behavioral Sciences, Seattle, Washington, and Texas A&M College of Medicine, Department of Psychiatry, Temple, Texas; Division of Adult and Community Health, Epilepsy Program, Centers for Disease Control and Prevention, Atlanta, Georgia; University of California Los Angeles School of Public Health, Center for Health Policy Research, Los Angeles, California; University of California Los Angeles School of Public Health, Center for Health Policy Research, Los Angeles, California

## Abstract

**Introduction:**

Epilepsy, which requires complex care, can be further complicated by comorbid mental illness. Evidence indicates deficiencies exist in the care received for both epilepsy-related care and for mental health care in people with epilepsy. Evidence indicates there are deficiencies in both these areas for people with epilepsy. Our objective was to evaluate treatment gaps in epilepsy and mental health care among California adults with epilepsy and to compare the mental health services and treatment received by people with epilepsy to that of the general population.

**Methods:**

We conducted multivariate analyses of data from the 2005 California Health Interview Survey (N = 43,020), which included data from 604 adult participants who said they had been told they had epilepsy, to examine comparisons of interest.

**Results:**

Twenty-seven percent of California adults with epilepsy who had had at least 1 seizure in the past 3 months had not seen a neurologist or epilepsy specialist in the past year. Of respondents with psychological distress and epilepsy, 84% perceived a need for mental health care in the past year, but only 57% had seen a mental health provider during that time. Of respondents without epilepsy but with psychological distress, 77% perceived a need for mental health care in the past year, but only 32% had seen a mental health provider during that time.

**Conclusion:**

California adults with epilepsy appear to have substantial unmet needs in both epilepsy care and mental health care. Adults with epilepsy and psychological distress appeared to have received more mental health treatment than psychologically distressed adults without epilepsy. Efforts should be made to improve access to quality epilepsy care that includes assessment and treatment of mental health disorders.

## Introduction

Epilepsy, a chronic neurological disorder, affects approximately 2 million Americans and is more common than autism, cerebral palsy, multiple sclerosis, and Parkinson disease combined (1). The likelihood of premature death increases with some types of epilepsy, especially if seizures are not well controlled (2). The risk for suicide among people with epilepsy is 5 times the rate of the national population, reflecting the burden of untreated mental illness in this population (3-5). People with epilepsy require complex care but often face barriers accessing quality treatment, which hinder care and increase health disparities (6-8). US population-based studies indicate that up to 36% of adults with epilepsy had not seen a neurologist or epileptologist in the past year, and as many as 26% of people with recent seizures did not take medicine to control seizures (7,9,10). *Healthy People 2020*, a set of national objectives to improve population health in the United States, includes a goal of increasing the proportion of people with epilepsy and uncontrolled seizures who receive appropriate medical care (11).

The Centers for Disease Control and Prevention (CDC) supported questions in the 2005 California Health Interview Survey (CHIS) to examine health services among community-dwelling adults with epilepsy. Our objective was to use the 2005 CHIS to examine treatment gaps in epilepsy and mental health care in California adults with epilepsy and to compare the mental health services and treatment received by people with epilepsy to that of the general population.

## Methods

CHIS is a geographically stratified, 2-stage telephone survey that has been conducted every other year since 2001. The data are cross-sectional. The first stage draws a sample of telephone numbers using a list-assisted random-digit–dial method; in the second stage, 1 adult (aged 18 or older) is randomly selected among all adults in the household as the respondent. The methods are described in detail elsewhere (9,12). In its 2005 data collection cycle, CHIS completed interviews with 43,020 adults statewide. Interviews were conducted in English, Spanish, Chinese, Korean, and Vietnamese. Detailed information on the complex CHIS 2005 weighting procedures is available at www.chis.ucla.edu/pdf/CHIS2005_method5.pdf. The response rate for the adult extended interview was 54% using the American Association for Public Opinion Research's fourth method for calculating response rate. Detailed information on CHIS response rates is available at www.chis.ucla.edu/pdf/CHIS2005_method4.pdf. Procedures for data collection and analysis were approved by the institutional review boards at the University of California, Los Angeles, and the California Health and Human Services Agency.

### Measures

Three CDC-developed questions were used to identify people with epilepsy and classify them as having active or inactive epilepsy (7): 1) “Have you ever been told by a doctor that you have a seizure disorder or epilepsy?,” 2) “Are you currently taking any medicine to control your seizure disorder or epilepsy?,” and 3) “How many seizures have you had in the last 3 months?” Respondents were defined as having active epilepsy if they reported being told by a physician that they had epilepsy and reported either taking a medicine to control epilepsy or having at least 1 seizure in the past 3 months. Active epilepsy was further categorized by whether there had been a seizure in the past 3 months (“current seizures”). This distinction is relevant because adults with active epilepsy and current seizures are a high-priority group (ie, refractory/uncontrolled epilepsy contrasted with active but well-controlled epilepsy), warranting intervention. Adults who had ever been told by a doctor that they had a seizure disorder or epilepsy (ie, a history of epilepsy) but were not taking medication for epilepsy and had not had a seizure in the past 3 months were classified as having inactive epilepsy (this group includes respondents who responded “no longer have epilepsy” to the question about seizures in the last 3 months) (7). The CDC-developed questions also ask the following: 1) “In the past 12 months, have you seen a neurologist or epilepsy specialist for your epilepsy and seizure disorder?” and 2) “During the past month, to what extent has your epilepsy and its treatment interfered with normal activities like working, school, or getting together with family and friends?” Respondents who answered “moderately,” “quite a bit,” or “extremely” were defined as having their epilepsy interfere with normal activities.

The presence of psychological distress was measured using the Kessler-6 (K6), an epidemiologic tool with good precision for identifying cases of DSM-IV (*Diagnostic and Statistical Manual of Mental Disorders, Fourth Edition*) disorders in the community (13). The K6 has 5 response options, scored from 0 to 4, that are summed across the 6 items to create a scale (range, 0-24); a score of 13 or more indicates psychological distress. The K6 is an efficient screen for "serious mental illness" in the general population (14).

All CHIS respondents were asked the following validated question from CDC's Healthy Days Measures: "Now thinking about your mental health, which includes stress, depression, and problems with emotions, for how many days during the past 30 days was your mental health not good?" (15). Respondents who said that their mental health was not good for 14 or more of the preceding 30 days were defined as having frequent mental distress (16).

To assess use of health services, respondents were asked whether they had health insurance currently, whether they had been insured all or part of the past year, whether they had a usual source of care, and whether they had met with a physician in the past year. Respondents were also asked about their perceived need for mental health care and their use of mental health services in the past 12 months. Whether health insurance covered mental health care was asked only of respondents who responded affirmatively to either of the 2 questions on perceived need for mental health care or use of mental health services. Psychotropic drug use was assessed with the question, "During the past 12 months, did you take any prescription medications, such as an antidepressant or sedative, almost daily for 2 weeks or more, for an emotional or personal problem?" Respondents who reported needing or receiving help for an emotional or mental health problem were asked, "During the past 12 months, did you have difficulties in getting mental health treatment?" Respondents were also asked, "In the past 12 months, did you receive care in an emergency room for emotional or mental health problems?"

### Statistical analysis

SAS version 9.1 (SAS Institute, Inc, Cary, North Carolina) and SAS-callable SUDAAN version 8.0 (RTI International, Research Triangle Park, North Carolina) were used for all analyses. Univariate statistics were produced for the sociodemographic health factors and measures of mental health use, separately for adults with and without a history of epilepsy, and nonoverlapping 95% confidence intervals were used to examine differences between groups. Logistic regression models were fit to estimate odds ratios for several outcome measures based on epilepsy history and mental distress while controlling for sociodemographic characteristics (age and sex) and the high prevalence of medical comorbidities (self-reported doctor-diagnosed asthma, diabetes, high blood pressure, heart disease, stroke, and cancer) in adults with epilepsy thought to confound associations with mental health outcomes of interest (7). In this small sample of adults with epilepsy, the introduction of other covariates (race/ethnicity, employment, insurance status) in the multivariate models reduced observations, in some cases to 1 person in each cell, precluding the use of these additional covariates. To produce accurate standard errors, replicate weights were used to control for the complex CHIS sample design. Additional information on the use of replicate weights with the CHIS data is available at www.chis.ucla.edu/pdf/weighting_var_chis_02282007.pdf

## Results

### Characteristics

Results from the 2005 CHIS (12,17) indicated that adults with a history of epilepsy were less likely to be married and less likely to have obtained a college or graduate degree than adults without a history of epilepsy. Significantly more respondents with a history of epilepsy had a physical disability or were unable to work and had psychological distress and frequent mental distress.

### Access to care and epilepsy-related activity limitations

Most adults in this survey (82%) had seen a physician in the past 12 months (Table 1). Compared with adults with inactive epilepsy, adults with active epilepsy were more likely to have seen a neurologist or epilepsy specialist in the past 12 months. Even so, an estimated 27% of those reporting a seizure in the past 3 months had not seen an epilepsy specialist or neurologist during the preceding year. Significantly more adults with active epilepsy and current seizures reported that epilepsy interfered with their normal activities, compared with those with active epilepsy but without current seizures. Current and past-year health insurance status did not differ significantly between respondents with active epilepsy, inactive epilepsy, and no epilepsy, but 10% to 21% of these groups were uninsured in 2005 (Table 1).

### Mental health care and psychological distress

Compared with respondents without epilepsy, those with active epilepsy (with or without current seizures) were more likely to have perceived a need for mental health care in the past 12 months (Table 1). Respondents with active epilepsy (with or without current seizures) and inactive epilepsy were more likely to have seen a mental health care provider in the past 12 months than were those without a history of epilepsy (Table 1). Respondents with active epilepsy with current seizures and inactive epilepsy were also more likely to have been to an emergency department for mental health care. After adjustment for age, sex, and medical comorbidities, adults with active epilepsy with current seizures were more likely to have taken a psychotropic drug for mental health care in the past 12 months than were those without a history of epilepsy (Table 2). No significant differences in reported delays were found for mental health treatment between those with epilepsy and the general population (Table 2).

More than three-fourths of all adults with psychological distress perceived a need for mental health care in the past year (Table 3). Compared with psychologically distressed people without epilepsy, psychologically distressed people with epilepsy were more likely to have seen a mental health provider in the last 12 months (57% vs 32%) (Table 3). Adults with psychological distress were more likely than those without such distress to have been to an emergency department for mental health care in the past year, but the percentages were small (Table 3). Almost 1 of 5 adults with epilepsy and psychological distress had been uninsured all or part of the previous year, while 32% of adults without epilepsy but with psychological distress reported the same experience (Table 3).

Adults with epilepsy, both with and without psychological distress, and adults without epilepsy but with psychological distress were more likely to have taken a psychotropic drug for mental health care in the past 12 months than were those with no history of epilepsy and no psychological distress (Table 3). Less than one-third of all adults with psychological distress had experienced delays in getting mental health treatment in the past 12 months (Table 3). Most adults with epilepsy and psychological distress (86%) and without epilepsy but with psychological distress (76%) had health insurance coverage that included mental health coverage (data not shown), meaning that 14% and 24% of these subgroups, respectively, had no mental health coverage.

## Discussion

The gap in epilepsy care is the difference between having everyone with active epilepsy see a neurologist or epilepsy specialist and the actual percentage who reported seeing such a physician. We found that approximately 27% of adult Californians with epilepsy and recent seizures had not seen a neurologist or epilepsy specialist in 12 months, a finding similar to those of other population-based studies. In the 2004 HealthStyles Survey, only 36% of respondents with self-reported epilepsy had seen a neurologist or epileptologist in the past 12 months (10); the 2005 Behavioral Risk Factor Surveillance System survey found that 16% of adults with epilepsy and recent seizures were not taking medicine to control their seizures, and 35% had not seen a neurologist or epileptologist in the past year (7).

These data may overstate the treatment gap. For example, most respondents to the 2005 CHIS with active epilepsy and current seizures had seen a physician in the past 12 months. These providers may have collaborative relationships with neurologists or epileptologists so that appropriate changes in epilepsy treatment are made without the patient seeing the neurologist. These data may also understate the difference between respondents who have epilepsy and those who receive any specialty treatment. The survey includes a general neurologist as an epilepsy specialist; this may not be sufficient, especially for those with treatment-resistant epilepsy.

We estimate, conservatively, that 40% of California adults with epilepsy and psychological distress do not receive mental health care, indicative of a second treatment gap. Population-based studies clearly demonstrate that people with epilepsy have more mental disorders than the general population (17-19). Furthermore, community-based studies indicate that people with epilepsy and psychiatric disorders often do not receive psychiatric care. One study found that 38.5% of depressed people with epilepsy had never been evaluated or treated for depression (20).

Our study suggests that psychologically distressed adults with epilepsy receive as much (perhaps more) mental health care than the general California population with psychological distress. This finding may be because adults with epilepsy are already in a health care system, facilitating access to other medical or mental health care, to the diligence of those who provide their epilepsy care, or California public policy that facilitates access to mental health treatment for some (21).

The deficiency in mental health treatment for people with epilepsy in our study may illustrate the general gap for mental health care that exists in the United States (22,23). Furthermore, although we found that more than half of adults with epilepsy and psychological distress saw a mental health provider in the past year and a somewhat larger percentage took a psychotropic drug in the past year, whether any of this represents quality mental health care is unknown. In the 2005 CHIS, seeing a mental health provider could mean seeing a therapist only once in the past year. If we had used a standard that one had to receive quality mental health care, the gap in services would be considerably larger.

A thorough effort (24) to define "quality" epilepsy care detailed these follow-up requirements for patients with this disorder: at least an annual review of epilepsy-specific topics such as drug-drug interactions, family planning, screening for mood disorders, interventions if a patient continues to have seizures or to experience side effects from medication, and mental health treatment or referral for specialty mental health care if a psychiatric disorder is diagnosed. These requirements suggest that, without dramatic changes in the resources available in primary care settings and the training received by the physicians, people with epilepsy need at least yearly contact with some member of a specialty epilepsy care team who can provide the education and screening needed to identify and treat morbidities. Furthermore, mental health care providers must collaborate with neurologists and epileptologists to directly and indirectly guide the mental health care treatment that will affect adherence to medication, seizure control, and quality of life. Finally, effective community-based interventions designed to treat comorbid mental illness in people with epilepsy can be implemented more broadly to improve their quality of life (25,26).

This study had several limitations. The data were self-reported and may be subject to recall bias. Our definition of active epilepsy differs from that provided by the International League Against Epilepsy, but it has been accepted for many large-scale surveillance studies (7). No causal associations can be drawn between epilepsy status and serious psychological distress because these data are cross-sectional. There may be misclassification of acute symptomatic seizures or nonepileptic seizures as epilepsy, although it is unlikely that the small percentage of adults estimated to have nonepileptic seizures would skew estimates (27). Stigma related to reporting epilepsy or psychological distress may have resulted in underreporting of these conditions (28). Cases of psychological distress may also have been underestimated because of effective treatment in some cases, but this is unlikely to substantially change findings, given the associations seen between mental health treatment variables in respondents with epilepsy and no distress. Although epilepsy data were available in 2003 and 2005, mental health–related variables were only available on the 2005 CHIS. Therefore, the data we used were limited to a small sample of respondents with epilepsy and with psychological distress, precluding additional reliable analysis of subgroups (eg, race, income, insurance status). Also, the small sample size resulted in some odds ratios that exaggerate the magnitude of associations between independent and dependent variables. Finally, although the use of imputation on some survey variables may be a limitation, there were few cases of imputation in this study, so the effects on these data would most likely be small.

This is the first large population-based study in the United States examining the mental health treatment gap for people with active epilepsy. Large numbers of adults with epilepsy perceive a need for mental health treatment, but fewer receive treatment from a mental health provider. This research supports the conclusion that quality epilepsy care must include the assessment for and treatment of mental health disorders. Additional population-based studies can be supported in other states and nationally to examine how patterns in access to mental health care for people with epilepsy may differ from those seen in California.

## Acknowledgments

We thank Dr David J. Thurman for his helpful review and thoughtful comments. During part of this project, Dr Thompson was supported by National Research Service Award grant no. T32 MH020021.

## Figures and Tables

**Table 1.** Health Care Use and Perceptions of Need and Activity Limitation, by Epilepsy Status, California Health Interview Survey, 2005^a^

**Variable**	Active Epilepsy With Current Seizures (n = 130)	Active Epilepsy Without Current Seizures (n = 202)	Inactive Epilepsy (n = 272)	No History of Epilepsy (n = 42,416)
**Epilepsy interference with normal activities?**				
Yes	68.6 (55.3-79.4)	21.2 (14.9-29.2)	3.6 (1.5-8.3)	NA
No	31.4 (20.6-44.7)	78.8 (70.8-85.0)	96.4 (91.7-98.4)	
Odds ratio^b^	60.8 (21.4-173.0)^c^	7.6 (3.1-18.6)^c^	1 [Reference]	
**Neurologist or epilepsy specialist visit in past 12 months?**				
Yes	72.8 (59.7-83.0)	40.9 (32.2-50.2)	6.0 (2.4-14.1)	NA
No	27.2 (17.1-40.2)	59.1 (49.8-67.8)	94.0 (85.9-97.6)	
Odds ratio^b^	41.3 (11.2-152.2)^c^	10.6 (3.1-35.6)^c^	1 [Reference]	
**Visits with a medical doctor in past 12 months?**				
Yes	93.4 (81.6-97.9)	95.6 (89.4-98.3)	85.0 (76.4-90.8)	81.6 (81.0-82.2)
No	6.6 (2.1-18.4)	4.4 (1.7-10.6)	15.0 (9.2-23.5)	18.4 (17.8-19.0)
Odds ratio^b^	2.7 (0.6-11.5)	4.3 (1.4-13.4)^c^	1.2 (0.6-2.3)	1 [Reference]
**Health insurance currently?**				
Yes	81.7 (61.0-93.0)	89.6 (82.5-94.1)	79.0 (69.5-86.1)	83.9 (83.3-84.5)
No	18.3 (7.2-39.0)	10.4 (5.9-17.5)	21.1 (13.9-30.6)	16.1 (15.5-16.7)
Odds ratio^b^	0.8 (0.3-2.0)	1.4 (0.7-2.7)	0.7 (0.4-1.2)	1 [Reference]
**Health insurance past year?**				
Insured all year	76.5 (56.8-89.0)	85.3 (77.4-90.8)	75.9 (66.5-83.4)	78.6 (78.0-79.2)
Uninsured all or part of year	23.5 (11.0-43.2)	14.7 (9.2-22.6)	24.1 (16.6-33.5)	21.4 (20.8-22.0)
Odds ratio^b^	0.9 (0.3-2.0)	1.4 (0.7-2.4)	0.9 (0.5-1.4)	1 [Reference]
**Usual source of care?**				
Yes	96.1 (90.6-98.5)	92.6 (84.2-96.7)	79.3 (69.9-86.3)	85.7 (85.2-86.2)
No	3.9 (1.5-9.4)	7.4 (3.3-15.7)	20.7 (13.7-30.1)	14.3 (13.8-14.9)
Odds ratio^b^	3.9 (1.2-11.7)^c^	1.7 (0.7-4.0)	0.6 (0.3-1.1)	1 [Reference]
**During the past 12 months, did you think you needed mental health care?**				
Yes	54.5 (39.2-69.0)	32.1 (24.2-41.2)	24.4 (17.4-33.1)	18.4 (17.8-19.0)
No	45.5 (31.0-60.8)	67.9 (58.8-75.8)	75.6 (66.9-82.6)	81.6 (81.0-82.2)
Odds ratio^b^	4.7 (2.2-9.5)^c^	2.1 (1.4-3.2)^c^	1.4 (0.9-2.1)	1 [Reference]
**Seen a mental health provider in the past 12 months?**				
Yes	28.6 (18.2-41.8)	22.4 (15.0-32.1)	17.3 (12.2-23.9)	8.2 (7.8-8.6)
No	71.4 (58.2-81.8)	77.6 (67.9-85.0)	82.7 (76.1-87.9)	91.8 (91.4-92.2)
Odds ratio^b^	3.9 (2.2-7.2)^c^	3.2 (1.9-5.3)^c^	2.3 (1.5-5.4)^c^	1 [Reference]
**Been to an emergency room for mental health care in the past 12 months?**				
Yes	5.3 (1.5-16.5)	1.5 (0.3-6.8)	1.5 (0.6-3.8)	0.4 (0.3-0.5)
No	94.7 (83.5-98.5)	98.5 (93.2-99.7)	98.5 (96.2-99.4)	99.6 (99.5-99.7)
Odds ratio^b^	10.6 (2.3-48.8)^c^	3.5 (0.2-50.1)	3.2 (1.1-9.3)^c^	1 [Reference]

Abbreviation: NA, not applicable.

a Values presented are % (95% confidence interval) unless otherwise indicated.

b Adjusted for age, sex, self-reported doctor-diagnosed asthma, diabetes, high blood pressure, heart disease, stroke, and cancer; the comparison looks at the response to the survey question across epilepsy status. For example, with the first question, the adjusted odds ratio for active epilepsy with current seizures is the odds a respondent with active epilepsy with current seizures reports epilepsy interferes with normal activities compared with a respondent with inactive epilepsy.

c Significant at *P* <.05; 95% confidence intervals calculated using logistic regression modeling.

**Table 2.** Mental Health Treatment, by Epilepsy Status, California Health Interview Survey, 2005^a^

**Mental Health Treatment**	Active Epilepsy With Current Seizures (n = 74)	Active Epilepsy Without Current Seizures (n = 73)	Inactive Epilepsy (n = 94)	No History of Epilepsy (n = 9,133)
**Taken a psychotropic drug for mental health care in the past 12 months?^b^**				
Yes	50.8 (32.6-68.8)	46.6 (30.9-63.0)	43.4 (30.7-56.9)	32.1 (30.6-33.5)
No	49.2 (31.2-67.4)	53.4 (37.0-69.1)	56.6 (43.1-69.3)	68.0 (66.5-69.4)
Odds ratio^c^	2.3 (1.1-4.5)^d^	1.7 (0.9-3.2)	1.5 (0.8-2.7)	1 [Reference]
**Did you have delays getting mental health treatment in past 12 months?^b^**				
Yes	7.4 (3.0-16.9)	14.2 (6.5-28.1)	11.7 (5.7-22.3)	6.4 (5.7-7.2)
No	92.7 (83.1-97.0)	85.8 (71.9-93.5)	88.3 (77.7-94.3)	93.6 (92.9-94.3)
Odds ratio^c^	1.1 (0.3-3.1)	2.3 (1.0-5.5)	1.8 (0.8-4.2)	1 [Reference]

a Values presented are % (95% confidence interval) unless otherwise indicated.

b Only asked of respondents reporting a perceived need or receipt of mental health care in the past 12 months.

c Adjusted for age, sex, self-reported doctor-diagnosed asthma, diabetes, high blood pressure, heart disease, stroke, and cancer; the comparison looks at the response to the survey question across epilepsy status. For example, with the first question, the adjusted odds ratio for active epilepsy with current seizures is the odds a respondent with active epilepsy with current seizures reports having taken a psychotropic for mental health care in the past 12 months compared to a respondent with inactive epilepsy.

d Significant at *P* <.05; 95% confidence intervals calculated using logistic regression modeling.

**Table 3.** Mental Health Care Services, Mental Health Treatment, and Psychological Distress, by Epilepsy Status, California Health Interview Survey, 2005^a^

Variable	All Respondents			

	History of Epilepsy		No History of Epilepsy	

	Psychologically Distressed (n = 83)	Not Distressed (n = 513)	Psychologically Distressed (n = 1,575)	Not Distressed (n = 40,708)
**During the past 12 months, did you think you needed mental health care?**				
Yes	84.2 (70.5-92.2)	26.7 (20.6-33.8)	77.1 (73.6-80.2)	16.1 (15.5-16.8)
No	15.8 (7.8-29.4)	73.3 (66.2-79.4)	23.0 (19.8-26.4)	83.9 (83.2-84.5)
Odds ratio^b^	27.3 (12.3-60.6)^c^	1.8 (1.2-2.6)^c^	17.9 (14.8-21.4)^c^	1 [Reference]
**Seen a mental health provider in the past 12 months?**				
Yes	56.7 (42.9-69.6)	16.8 (13.6-20.6)	32.3 (28.8-35.9)	7.2 (6.9-7.6)
No	43.3 (30.4-57.2)	83.2 (79.4-86.4)	67.7 (64.1-71.2)	92.8 (92.4-93.2)
Odds ratio^b^	NA	2.5 (1.9-3.2)^c^	5.8 (4.8-6.9)^c^	1 [Reference]
**Been to an ER for mental health care in the past 12 months?**				
Yes	9.2 (3.6-21.7)	1.5 (0.5-4.1)	4.8 (3.5-6.6)	0.3 (0.1-0.4)
No	90.8 (78.3-96.4)	98.5 (96.0-99.5)	95.2 (93.4-96.5)	99.7 (99.6-99.9)
Odds ratio^b^	34.4 (10.9-107.6)^c^	5.6 (1.5-19.9)^c^	18.9 (12.6-28.5)^c^	1 [Reference]
**Health insurance in past 12 months?**				
Insured all year	81.5 (70.4-89.2)	78.2 (71.1-84.0)	68.5 (64.8-72.0)	78.9 (78.2-79.6)
Uninsured all or part year	18.5 (10.8-29.6)	21.8 (16.0-28.9)	31.5 (28.0-35.2)	21.1 (20.4-21.8)
Odds ratio^b^	0.89 (0.44-1.81)	0.94 (0.6-1.4)	0.5 (0.3-0.6)^c^	1 [Reference]

**Variable**	**Respondents Reporting a Perceived Need or Receipt of Mental Health Care in the Past 12 Months**			

	**History of Epilepsy**		**No History of Epilepsy**	

	**Psychologically Distressed (n = 69)**	**Not Distressed (n = 172)**	**Psychologically Distressed (n = 1,279)**	**Not Distressed (n = 7,854)**

**Taken a psychotropic for mental health care in the past 12 months?**				
Yes	62.1 (43.4-78.0)	41.9 (31.3-53.3)	49.2 (45.1-53.2)	29.2 (27.8-30.7)
No	37.9 (22.1-56.6)	58.1 (46.8-68.7)	50.8 (46.8-54.9)	70.8 (69.3-72.2)
Odds ratio^b^	3.4 (1.5-7.5)^c^	1.7 (1.1-2.7)^c^	2.1 (1.8-2.6)^c^	1 [Reference]
**Did you have delays getting mental health treatment in past 12 months?**				
Yes	29.8 (16.8-47.0)	5.3 (3.0-9.1)	18.7 (15.6-22.3)	4.4 (3.7-5.1)
No	70.3 (53.0-83.2)	94.7 (90.9-97.0)	81.3 (77.8-84.4)	95.7 (94.9-96.3)
Odds ratio^b^	8.8 (4.0-19.3)^c^	1.2 (0.6-2.2)	5.0 (3.7-6.8)^c^	1 [Reference]

Abbreviations: NA, not available (sample size too small for reliable estimates); ER, emergency room.

a Values presented are % (95% confidence interval) unless otherwise indicated.

b Adjusted for age, sex, doctor-diagnosed asthma, diabetes, high blood pressure, heart disease, stroke, and cancer; the comparison looks at the response to the survey question across epilepsy status category. For example, with the first question, the adjusted odds ratio for a psychologically distressed person with epilepsy is the odds a respondent with history of epilepsy and psychological distress reported a perceived need for mental health care in the past 12 months compared to a person without a history of epilepsy or psychological distress.

c Significant at *P* <.05; 95% confidence intervals calculated using logistic regression modeling.

## References

[1] Hirtz D, Thurman DJ, Gwinn-Hardy K, Mohamed M, Chaudhuri AR, Zalutsky R (2007). How common are the "common" neurologic disorders?. Neurology.

[2] Tomson T, Walczak T, Sillanpaa M, Sander JW (2005). Sudden unexpected death in epilepsy: a review of incidence and risk factors. Epilepsia.

[3] Rafnsson V, Olafsson E, Hauser WA, Gudmundsson G (2001). Cause-specific mortality in adults with unprovoked seizures. A population-based incidence cohort study. Neuroepidemiology.

[4] Wiegartz P, Seidenberg M, Woodard A, Gidal B, Hermann B (1999). Co-morbid psychiatric disorder in chronic epilepsy: recognition and etiology of depression. Neurology.

[5] Boylan LS, Flint LA, Labovitz DL, Jackson SC, Starner K, Devinsky O (2004). Depression but not seizure frequency predicts quality of life in treatment-resistant epilepsy. Neurology.

[6] Theodore WH, Spencer SS, Wiebe S, Langfitt JT, Ali A, Shafer PO (2006). Epilepsy in North America: a report prepared under the auspices of the Global Campaign against Epilepsy, the International Bureau for Epilepsy, the International League Against Epilepsy, and the World Health Organization. Epilepsia.

[7] Kobau R, Zahran H, Thurman DJ, Zack MM, Henry TR, Schachter SC (2005). Epilepsy surveillance among adults — 19 states, Behavioral Risk Factor Surveillance System, 2005. MMWR Surveill Summ.

[8] Szaflarski M, Szaflarski JP, Privitera MD, Ficker DM, Hormer RD (2006). Racial/ethnic disparities in the treatment of epilepsy: what do we know? What do we need to know?. Epilepsy Behav.

[9] Kobau R, Zahran H, Grant D, Thurman DJ, Price PH, Zack MM (2007). Prevalence of active epilepsy and health-related quality of life among adults with self-reported epilepsy in California: California Health Interview Survey, 2003. Epilepsia.

[10] Kobau R, Gilliam F, Thurman DJ (2006). Prevalence of self-reported epilepsy or seizure disorder and its associations with self-reported depression and anxiety: results from the 2004 HealthStyles Survey. Epilepsia.

[11] Healthy people 2020. Disability and health objectives: systems and policies, barriers to health care, objective DH-6.

[12] Elliott JO, Lu B, Moore JL, McAuley JW, Long L (2008). Exercise, diet, health behaviors, and risk factors among persons with epilepsy based on the California Health Interview Survey, 2005. Epilepsy Behav.

[13] Kessler RC, Andrews G, Colpe LJ, Hiripi E, Mroczek DK, Normand SL (2002). Short screening scales to monitor population prevalences and trends in non-specific psychological distress. Psychol Med.

[14] Kessler RC, Barker PR, Colpe LJ, Epstein JF, Gfroerer JC, Hiripi E (2003). Screening for serious mental illness in the general population. Arch Gen Psychiatry.

[15] Moriarty DG, Zack MM, Kobau R (2003). The Centers for Disease Control and Prevention's Healthy Days Measures — population tracking of perceived physical and mental health over time. Health Qual Life Outcomes.

[16] (1998). Self-reported frequent mental distress among adults — United States, 1993-1996. MMWR Morb Mortal Wkly Rep.

[17] Moore JL, Elliott JO, Lu B, Klatte ET, Charyton C (2009). Serious psychological distress among persons with epilepsy based on the 2005 California Health Interview Survey. Epilepsia.

[18] Tellez-Zenteno JF, Patten SB, Jette N, Williams J, Wiebe S (2007). Psychiatric comorbidity in epilepsy: a population-based analysis. Epilepsia.

[19] Strine TW, Kobau R, Chapman DP, Thurman DJ, Price P, Balluz LS (2005). Psychological distress, comorbidities, and health behaviors among US adults with seizures: results from the 2002 National Health Interview Survey. Epilepsia.

[20] Ettinger A, Reed M, Cramer J (2004). Depression and comorbidity in community-based patients with epilepsy or asthma. Neurology.

[21] Mental Health Services Act (Proposition 63).

[22] Regier DA, Narrow WE, Rae DS, Manderscheid RW, Locke BZ, Goodwin FK (1993). The de facto US mental and addictive disorders service system. Epidemiologic catchment area prospective 1-year prevalence rates of disorders and services. Arch Gen Psychiatry.

[23] Kessler RC, Demler O, Frank RG, Olfson M, Pincus HA, Walters EE (2005). Prevalence and treatment of mental disorders, 1990 to 2003. N Engl J Med.

[24] Pugh MJ, Berlowitz DR, Montouris G, Bokhour B, Cramer JA, Bohm V (2007). What constitutes high quality of care for adults with epilepsy?. Neurology.

[25] Ciechanowski P, Chaytor N, Miller J, Fraser R, Russo J, Unutzer J, Gilliam F (2010). PEARLS Depression Treatment for Individuals with Epilepsy: a randomized controlled trial. Epilepsy Behav.

[26] Thompson NJ, Reisinger Walker E, Obolensky N, Winning A, Barmon C, DiIorio C, Compton M (2010). Distance delivery of mindfulness-based cognitive therapy for depression: Project UPLIFT. Epilepsy Behav.

[27] Alsaadi TM, Marquez AV (2005). Psychogenic nonepileptic seizures. Am Fam Physician.

[28] Jacoby A (2002). Stigma, epilepsy, and quality of life. Epilepsy Behav.

