# Erratum, Vol. 9, Feb. 23 Release

**Published:** 2012-04-05

Because of an editing error, a percentage was reported incorrectly in the article "Epilepsy Care and Mental Health Care for People with Epilepsy: California Health Interview Survey, 2005." The sentence that read "We found that approximately 73% of adult Californians with epilepsy and recent seizures had not seen a neurologist or epilepsy specialist in 12 months, a finding similar to those of other population-based studies," should have read, "We found that approximately 27% of adult Californians with epilepsy and recent seizures had not seen a neurologist or epilepsy specialist in 12 months, a finding similar to those of other population-based studies." This correction was made to our website on March 20, 2012, and appears online at http://www.cdc.gov/pcd/issues/2012/11_0140.htm. We regret any confusion or inconvenience this error may have caused.

